# Commentary on: “Impact of Nimodipine-Induced Blood Pressure Reductions on Cerebral Autoregulation and Functional Outcome after Aneurysmal Subarachnoid Hemorrhage”

**DOI:** 10.1007/s12028-026-02488-1

**Published:** 2026-03-26

**Authors:** Michael Veldeman

**Affiliations:** Department of Neurosurgery, RWTH Aachen University Hospital, Pauwelsstrasse 30, 52074 Aachen, Germany

In their observational cohort study, the authors examine the impact of nimodipine-induced blood pressure (BP) reductions on cerebral autoregulation and functional outcome after aneurysmal subarachnoid hemorrhage (aSAH), applying contemporary neuromonitoring to a therapy that has remained largely unchanged for decades [1]. We commend the authors for revisiting an established cornerstone of aSAH management through the lens of individualized autoregulatory physiology.

Nearly four decades ago, randomized controlled trials by Allen et al. and Pickard et al. established nimodipine as the only pharmacological intervention shown to improve outcome after aSAH [2, 3]. In the initial trial by Allen et al., severe neurological deficits due to cerebral arterial spasm or death within 21 days occurred in 13.3% of placebo-treated patients compared with 1.8% of those receiving nimodipine, corresponding to an absolute risk reduction of 11.5% and a number needed to treat of approximately nine [2]. In the larger British aneurysm nimodipine trial, Pickard et al. demonstrated a reduction in cerebral infarction from 33% to 22% (absolute risk reduction 11%) and a reduction in poor outcome (death or severe disability) from 33% to 20% (absolute risk reduction 13%), again yielding a number needed to treat of approximately eight to nine [3]. These trials firmly established nimodipine’s benefit at the population level, albeit with a modest absolute effect size.

Importantly, these landmark studies were conducted in an era that predated continuous bedside assessment of cerebral autoregulation and did not systematically quantify hypotension or its physiological consequences. A recent review also questioned the foundation of current nimodipine regimens and underscored persisting uncertainties regarding its optimal route, dose, and timing of administration [4]. In particular, pharmacokinetic data reveal large interindividual variability related to bioavailability, metabolism, and formulation differences between oral, enteral, and intravenous preparations. The present study addresses a clinically relevant gap by integrating near-infrared-spectroscopy-derived autoregulatory indices to derive individualized lower limits of autoregulation (LLA) [1, 5]. The authors’ observation that nimodipine-associated BP reductions may exceed personalized autoregulatory capacity in a subset of patients, and that time spent below the LLA correlates with worse 90-day functional outcome, provides important insight into potential heterogeneity of treatment effect.

We agree with the authors’ central implication that nimodipine’s benefit should not be questioned, but rather refined. As the authors show, the magnitude of BP reduction alone was less predictive of outcome than whether BP crossed an individual’s autoregulatory threshold. This distinction supports a shift from uniform BP targets toward physiology-guided management in selected patients, particularly those with impaired autoregulation.

Several methodological aspects appropriately reflect the exploratory nature of this work and can guide future studies. Exposure metrics were limited to the hour following nimodipine administration, representing only a fraction of total monitored time. Whether patients who accrue more post-dose time below the LLA also demonstrate a greater global burden of autoregulatory failure remains uncertain. Future analyses partitioning nimodipine-associated and non-nimodipine-associated periods or incorporating total time below the LLA as a covariate may further clarify causality. In addition, exclusion of administrations with limited optimal mean arterial pressure (MAPopt) availability raises the possibility of informative missingness if these periods coincided with greater physiological instability. Finally, the modest sample size necessarily limits multivariable adjustment, underscoring the need for prospective validation.

Beyond dosing strategies, the route of nimodipine administration warrants reconsideration in the context of individualized care. A Cochrane review comparing oral and intravenous nimodipine found no clear difference in efficacy with respect to infarction, poor outcome, or mortality, suggesting that intravenous administration may be an equally effective prophylactic option [6]. Intravenous nimodipine offers the practical advantage of continuous titration and rapid dose adjustment in patients who develop BP reductions below their autoregulatory limits. However, it should be noted that intravenous formulations contain ethanol as a solubilizing agent, which may exert independent biological effects and should be considered when interpreting physiological responses.

It is also important to distinguish prophylactic nimodipine therapy from its use as rescue treatment. Intra-arterial nimodipine has been employed as a last-tier intervention for delayed cerebral ischemia refractory to induced hypertension, with observational data demonstrating acceptable safety and functional independence in a substantial proportion of severely affected patients [7]. This application serves a fundamentally different therapeutic goal and should not be conflated with prophylactic dosing strategies aimed at outcome prevention.

Mechanistic insights further support individualized approaches. Experimental data suggest that nimodipine reduces post-hemorrhagic microvasospasms without affecting non-spastic microvessels, implicating microvascular dysfunction as a potential target of its neuroprotective effect [8]. If nimodipine’s benefit is mediated, at least in part, at the microcirculatory level, excessive systemic hypotension may counteract this effect by precipitating cerebral hypoperfusion in patients with impaired autoregulation.

In summary, this study represents an important step toward refining, rather than challenging, a foundational therapy in aSAH. Nimodipine confers a modest but reproducible benefit at the population level; modern neuromonitoring now offers the opportunity to identify patients in whom standard dosing may be suboptimal or potentially harmful. Autoregulation-guided strategies, individualized dosing, and alternative routes of administration merit prospective evaluation to preserve nimodipine’s neuroprotective effects while minimizing risk in vulnerable patients. We view this work as a constructive contribution that advances nimodipine therapy into the era of physiology-guided critical care.

## Author Compliance

I confirm that this manuscript complies fully with all instructions to authors provided by *Neurocritical Care*.

## Authorship Confirmation

I confirm that I meet all criteria for authorship as outlined by the International Committee of Medical Journal Editors (ICMJE), and that I have approved the final version of the manuscript prior to submission. As the sole author, I take full responsibility for the content of this work.
